# Data management challenges for artificial intelligence in plant and agricultural research

**DOI:** 10.12688/f1000research.52204.2

**Published:** 2023-01-17

**Authors:** Hugh F. Williamson, Julia Brettschneider, Mario Caccamo, Robert P. Davey, Carole Goble, Paul J. Kersey, Sean May, Richard J. Morris, Richard Ostler, Tony Pridmore, Chris Rawlings, David Studholme, Sotirios A. Tsaftaris, Sabina Leonelli

**Affiliations:** 1 Exeter Centre for the Study of the Life Sciences & Institute for Data Science and Artificial Intelligence, University of Exeter, Exeter, UK; 2 Department of Statistics, University of Warwick, Coventry, UK; 3 NIAB, National Research Institute of Brewing, East Malling, UK; 4 Earlham Institute, Norwich, UK; 5 Department of Computer Science, University of Manchester, Manchester, UK; 6 Royal Botanic Gardens, Kew, UK; 7 School of Biosciences, University of Nottingham, Loughborough, UK; 8 John Innes Centre, Norwich, UK; 9 Department of Computational and Analytical Sciences, Rothamsted Research, Harpendem, UK; 10 School of Computer Science, University of Nottingham, Nottingham, UK; 11 Biosciences, University of Exeter, Exeter, UK; 12 Institute of Digital Communications, University of Edinburgh, Edinburgh, UK; 13 Alan Turing Institute, London, UK

**Keywords:** data science, plant science, crop science, agricultural research, machine learning, data management, data quality, data sharing

## Abstract

Artificial Intelligence (AI) is increasingly used within plant science, yet it is far from being routinely and effectively implemented in this domain. Particularly relevant to the development of novel food and agricultural technologies is the development of validated, meaningful and usable ways to integrate, compare and visualise large, multi-dimensional datasets from different sources and scientific approaches. After a brief summary of the reasons for the interest in data science and AI within plant science, the paper identifies and discusses eight key challenges in data management that must be addressed to further unlock the potential of AI in crop and agronomic research, and particularly the application of Machine Learning (AI) which holds much promise for this domain.

## 1. Introduction

Data science is central to the development of plant and agricultural research and its application to social and environmental problems of a global scale, such as food security, biodiversity and climate change. Artificial Intelligence (AI) offers great potential towards elucidating and managing the complexity of biological data, organisms and systems. It constitutes a particularly promising approach for the plant sciences, which are marked by the distinctive challenge of understanding not only complex gene-environment (GxE) interactions that span multiple scales from the cellular through the microbiome to climate systems, but also their interaction with rapidly shifting human management practices (GxExM) in agricultural and other settings, whose reliance on digital innovations is growing at a fast pace ( Wang et al. 2020; Harfouche et al. 2019). Accordingly, examples of useful applications of AI – and particularly Machine Learning (ML) – to plant science contexts are increasing, with the Covid-19 pandemic crisis further accelerating interest in this approach ( King 2020).

Nevertheless, we are still far from a research landscape in which AI can be routinely and effectively implemented. A key obstacle concerns the development and implementation of effective and reliable data management strategies. Developing reliable and reproducible AI applications depends on having validated, meaningful and usable ways to integrate large, multi-dimensional datasets from different sources and scientific approaches. This is especially relevant to the development of novel food and agricultural technologies, which rely on research from diverse fields including fundamental plant biology, crop research, conservation science, soil science, plant pathology, pest/pollinator ecology and management, water and land management, climate modelling, agronomy and economics.

This paper explores data-related challenges to potential applications of AI in plant science, with particular attention paid to the analysis of GxExM interactions of relevance to crop science and agricultural implementations. It brings together the experiences of an interdisciplinary set of researchers from the plant and agricultural sciences, the engineering and computational sciences and the social studies of science, all of whom are working with complex datasets spanning genomic, physiological and environmental data and computational methods of analysis. The first part of the paper provides a brief overview of contemporary AI and data science applications within plant science, with particular attention paid to the UK and European landscape where the authors are based. The second part identifies and discusses eight challenges in data management that must be addressed to further unlock the potential of AI for plant science and agronomic research. We conclude with a reflection on how transdisciplinary and international collaborations on data management can foster impactful and socially responsible AI in this domain.

## 2. AI in plant research: current status and challenges

Following wider trends in the biosciences, both basic and applied plant sciences have increasingly emphasised data-intensive modes of research over the last two decades ( Leonelli et al. 2017; Leonelli 2016, 2019). The capacity to measure biological complexity at the molecular, organismal and environmental scales has increased dramatically, as demonstrated by: advances in high-throughput genomics and norms and tools that have supported the development of a commons of publicly shared genomic data; the development of platforms for high-throughput plant phenotyping in the laboratory, the greenhouse and the field; and the proliferation of remote sensing devices on-farm ( Tardieu et al. 2017). Such platforms and associated data generation have contributed to a booming AI industry in commercial agriculture, focused on the delivery of “precision” farming strategies, with estimates that the market will be worth US$1.55 billion by 2025. ^1^ Indeed, AI applications in plant research and agriculture have so far primarily benefited large-scale industrial farming ( Carbonell 2016), with R&D investment focused on commodity crops such as wheat, rice and maize; high-value horticulture crops such as soft fruits; and the enhancement of large-scale orchards and vineyards. In addition to this, however, the amount and type of data being collected, alongside advancements in AI methods, offer the opportunity to ask and address new questions of great importance to plant scientists and agricultural stakeholders around the world ( Tsiligiridis & Ainali 2018).

AI is the field of study and development of computer hardware and software that perform functions, such as problem-solving or learning, which have traditionally been considered properties of intelligent life. A range of research fields have contributed to the development of AI, currently the most prominent of which is machine learning (ML), the design of algorithms for data processing, prediction and decision support that are able to learn from *a priori* (“supervised”), inductive (“unsupervised”), and reward-based (“reinforcement”) experience ( Mitchell 1997). ^2^ This approach is particularly significant for applications that do not require an exact understanding of how the algorithm has reached its decision, as long as it has predictive power and it is possible to reproduce it ( Napoletani et al. 2015).

ML has been the dominant AI technology applied to plant and agricultural research so far. Many successful examples come from bioinformatics, where researchers may not need to worry about why a sequence of amino acids was classified as alpha-helical in structure as long as we know how reliable that prediction is, for instance. Indeed, ML has been widely used in the analysis of sequence data, for example to identify signal peptides and functional domains in amino-acid sequences via neural nets and profile hidden Markov models, such as Pfam and SMART ( El-Gebali et al. 2019, and see Larrañaga et al. 2006 for other classic examples). One key example from genomics that goes back to the 1990s is the use of models to identify genes and predict their functions based on training data from multiple species ( Hayes & Borodowski 1998; Birney et al. 2004; Zou et al. 2019). This has ongoing relevance for orphan and non-model crop research, where experimental approaches such as CRISPR knockouts to identify and validate gene function for individual species may not be feasible or cost-effective, but results may be inferred from experiments in model species ( Zou et al. 2019). Other challenges in genomics that can be addressed include the inference of gene regulatory networks ( Mochida et al. 2018) and the identification of pathogen virulence effector genes from genomic sequence data ( Sperschneider 2019), for example. Thus, ML can help to identify correlations not readily picked up by more traditional approaches and in turn suggest fruitful directions for further research. To date, whether or not correlations have biological meaning typically needs to be ascertained via experiment and/or observational data ( Leonelli 2014; Smith & Cordes 2019). Efforts towards explainable AI are, however, gaining momentum and both methodological and computational techniques are emerging which promise to support biological use of ML ( Schramowski et al. 2020).

Alongside applications in genomics, AI offers new opportunities for linking genotypes to phenotypes ( Wang et al. 2020). Image-based plant phenotyping has proven a particularly fertile area for the application of ML techniques, with the rapid development of non-destructive methods for the evaluation of plant responses to biotic and abiotic stress ( Singh et al. 2016; Mohanty et al. 2016; Ramcharan et al. 2017) and estimation of photosynthetic capacity ( Fu et al. 2019), as well as a variety of feature detection, counting, classification, and semantic segmentation tasks ( Jiang & Li 2020). With the arrival of deep supervised convolutional networks, progress in the performance of ML algorithms in predicting leaf counts increased considerably ( Dobrescu et al. 2017). Convolutional Neural Networks (CNNs) were also shown to be capable of performing challenging tasks of point feature detection ( Pound et al. 2018) and pixelwise segmentation ( Yasrab et al. 2019, Soltaninejad et al. 2020) on both roots and shoots in a variety of imaging modalities in both laboratory and field environments ( Gao et al. 2020). These technologies pose substantial new opportunities for analysing and understanding GxExM interactions through the integration of high throughput phenotyping data with other forms of research data, including genomic, field evaluation and climatic data. As well as addressing fundamental research questions, AI applications in this area offer the opportunity to understand and improve a range of practical activities from crop breeding through agricultural management (see Boxes 1 to 3). ^3^

Box 1. **AI opportunities: genomic selection.**

Genomic Selection (GS) is an approach for estimating breeding values for individual plants that can guide breeders’ decisions for selection and crossing ( Crossa et al. 2017), based on modelling associations between quantitative traits and a genome-wide set of markers. Accuracy of predictive models for GS and rate of genetic gain can be increased by employing ML, although the utility of ML in comparison to existing statistical models vary depending on the characteristics of the trait of interest ( Gonzalez-Camacho et al. 2018). A promising opportunity for the improvement of GS lies in using ML for the integration and the analysis of data from different omics layers (such as proteomics, metabolomics, metagenomics) that mediate between genotype and phenotype, facilitating the prediction of quantitative traits based on biological mechanisms rather than genetic marker associations and thereby increasing the reliability and utility of models for a wider range of populations than is currently possible ( Harfouche et al. 2019).

Box 2. **AI opportunities: long term experiments.**

Long-term experiments (LTE), where the same crop or crop rotation is grown for many years subject to a range of different management or treatment options, have an important place in agricultural research. Data from these experiments enable separation of agronomic and environmental (weather) influences on crop yield, and soil health over time and have done much to influence modern farming practices (e.g. Poulton et al. 2018; Jensen et al. 2020). The "Classical Experiments" at Rothamsted Research ( Parolini 2015, Macdonald et. al. 2018) are important examples. The data from these experiments, some of which were started in 1843, are available and documented in the e-RA data resource ( Perryman et al. 2018). Data from LTEs continue to be the subject of new analytical methods (e.g. Addy et al. 2020), yet remain a relatively untapped resource for knowledge discovery, in part because of the complexity of the experimental designs and the difficulty in accounting properly for the changes that might have occurred during their lifespans. To make LTEs more accessible for knowledge discovery, a recent initiative was launched by the Global Long Term Experiment Network to catalogue LTEs using a standard meta-data schema. The use of ML methods combining data from LTEs with local weather data might, for example, reveal hidden patterns in the data linked to long-term or higher order interactions within the data which could provide useful insights into the impact of future climate change.

Box 3. **AI opportunities: agricultural monitoring.**

AI offers many opportunities to improve the cost and labour efficiency of longstanding research and monitoring tasks in research and agricultural settings. While such possibilities are most developed in commercial agricultural settings, there are many opportunities too for the public research sector as well as for small or non-commercial farmers, for example in agricultural settings where there is limited access to relevant scientific expertise.

Example (1): Assessing soil health is a key driver of crop yields, yet wet soil chemistry analyses are both expensive and time-consuming and generally not accessible by growers in low and middle-income countries. Using near-infrared (NIR) and mid-infrared (MIR) soil spectroscopy data, ML models can be developed to predict soil characteristics and nutrient content that are faster and cheaper to run ( Data Study Group Team 2020). Such models could be integrated with plant physiology models in the future to predict optimal crop performance in a given soil, and open the possibility of the development of hand-held soil devices for use directly by farmers or local advisors in countries where lab access and resources are limited.

Example (2): Conventional methods such as suction and light traps for monitoring the appearance and migration of airborne insects, including crop pests, which currently need manual identification can also be augmented by ML models trained to recognise and classify insect species based on bioacoustic data (e.g. Potamitis et al. 2015, and under development by the Rothamsted Insect Survey), connected to in-field sonic sensors. Such developments are directed at increasing the scalability of the insect pest monitoring networks and also potentially removing the need for manual steps for some insect species.

Nevertheless, the effective implementation of AI in plant and agricultural science depends in large measure on establishing a favourable data landscape, consisting of the networks and practices of sourcing, managing and maintaining data. This is particularly important for research undertaken outside of resource-intensive commercial sites, including research in and for the Global South. Identifying the primary challenges faced by users and would-be users of AI in the contemporary data landscape of plant science is necessary in order to understand the possibilities and limitations afforded by AI for public as well as private plant and agricultural research. Here we build on the experiences of leading UK-based researchers in these areas to identify and discuss eight key data challenges, summarised in Table 1. These challenges span technical, social and governmental domains, and will require concerted, international and transdisciplinary efforts from a range of stakeholders to address. In the remainder of the paper, we review these challenges in detail, drawing on a range of examples from fundamental and translational plant science. Several of the challenges are shared with the biosciences more broadly, reflecting the conditions and complexity of biological research, while others are specific to plant science and agriculture. In the conclusion, we offer some reflections on how these challenges could be overcome.

**Table 1.** Synoptic view of the data challenges, possible solutions and what can be lost and gained by investment in those areas.

Data challenges	Solutions	Risks	Payoff	Trade-offs
*Heterogeneity of data types and sources in biology and agriculture*	Implement FAIR principles for all data types. Acknowledge and reward data sources.	Inconsistent standardisation between domains and communities.	New possibilities for multi-scale analysis integrating diverse data types.	Difficulties in implementing standards while retaining domain-specific insights.
*Selection and digitisation of data that is viable for AI applications*	Clear and accessible guidance on data requirements for AI. New procedures for priority setting and selecting data.	High labour costs of digitisation and analysis on resources that may not prove to be significant.	AI tools and outputs that push forward the cutting edge of plant science research.	Data management procedures may take up a considerable budget and effort.
*Ensuring sufficient linkage between biological materials and data used for AI applications*	Clear documentation of material provenance when producing data and throughout analytical workflows.	Increased documentation costs. Exposure of commercially or otherwise sensitive materials.	Clear understanding of the biological scope of AI tools.	Analysis of documentation around materials requires specific expertise and effort.
*Standardisation and curation of data and related software to a level appropriate for AI applications*	Development and use of shared semantic standards. Standardisation of data at the point of collection.	Potential to lose system-specific information that does not fit common standard.	Reusable multi-source data sets. Easier validation and sharing between groups.	Some plant data (e.g. phenotypic observations) remain very difficult to standardise.
*Obtaining training and adequate ground truth data for model validation and development*	Ensuring that data quality benchmarking is tailored to analytical purposes. Expanded collections of ground truth and training datasets.	Data quality assessment requires error estimates and information on data collection, which are often lacking.	Reproducible and sound inferences with clear scope of validity.	Tailoring data to specific research goals runs counter popular narrative of AI relying on ‘representative’ training data and ‘generalizable’ solutions.
*Access to and use of computing and modeling platforms, and related expertise*	Making software and models open and adaptable where appropriate, and/or have clear documentation on their scope. Provide researchers with full workflows, not only software.	Software used outside its range of proven usefulness. Danger of extrapolation and overfitting.	A suite of tools with clearly marked utility and relevance for a wide range of analytical tasks in the plant sciences.	Difficulties in getting the required know-how travel together with software and models.
*Improving responsible data access*	Opening access to datasets held by government and research institutions. Implementation of data governance regimes to protect sensitive data and ensure benefit sharing.	“Digital feudalism”; unequal distribution of benefits from public or personal data.	Greater data resources of direct relevance to agricultural and other plant science applications.	Ongoing difficulties in identifying and implementing non-exploitative, equitable models for data sharing.
*Engagement across plant scientists, data scientists, and other stakeholders*	Investment in and promotion of data services for plant scientists. Promotion of plant science problems, especially GxE interactions, to ML researchers. Identification of and investment in grand challenges and engagement.	High cost with potentially limited impact unless closely targeted to needs and interests of researchers and wider stakeholders.	Greater community participation in the development of ML as a resource for plant science.	Long term investment whose value depends on active and regular engagement of stakeholders.

**Table 2.** Examples of machine learning and artificial intelligence applications in plant and agricultural science discussed in this paper and methods used in those papers.

Example	Section discussed	Key ML/AI methods used	Sources
Gene identification and function prediction across species	2	Various; see citations in review paper	Zou et al. 2019
Inference of gene regulatory networks	2	Bayesian networks, random forest, Markov random fields, tree-based models, dynamic factor graph models	Mochida et al. 2018
Identification of pathogen virulence effector genes from genomic sequence data	2	Support vector machine, random forest, convolutional neural networks, ensemble learning, Bayesian networks, tree-based models	Sperschneider 2019
Non-destructive evaluation of plant responses to biotic and abiotic stress	2	Support vector machine, artificial neural networks, convolutional neural networks	Singh et al. 2016; Mohanty et al. 2016; Ramcharan et al. 2017
Automatic estimation of photosynthetic capacity	2	Artificial neural networks, support vector machine, least absolute shrinkage and selection operator (LASSO), random forest, Gaussian process regression	Fu et al. 2019
Convolutional neural networks for plant phenotyping image analysis	2	Convolutional neural networks, support vector machine, random forest, encoder-decoder model, multi-loss multi-resolution network, deep residual network	Jiang & Li 2020; Dobrescu et al. 2017; Pound et al. 2017; Yasrab et al. 2019; Soltaninejad et al. 2020
Augmenting Genomic Selection models in plant breeding with machine learning	Box 1	Bayesian regularized neural networks, radial basis function neural networks, reproducing kernel Hilbert space, random forest regression	Gonzalez-Camacho et al. 2018; Harfouche et al. 2019
Prediction of soil characteristics from near-infrared and mid-infrared soil spectroscopy data	Box 3	Regularised linear models, support vector mechanics, tree-based models	Data Study Group Team 2020
Automatic identification of crop pest insects using bioacoustics data	Box 3	Support vector machines, random forest, randomized trees classifier, gradient boosting classifier	Potamitis et al. 2015
Automatic digitisation of herbaria specimens and specimen metadata	3.2	Convolutional neural networks	Carranza-Rojas et al. 2017; Younis et al. 2018
Leaf-counting models for plant phenotyping image analysis	2, 3.6	Multi-task learning, adversarial learning, layerwise relevance propagation, guided back propagation	Dobrescu et al. 2017, 2019, 2020; Giuffrida et al. 2019
Computer Vision Problems in Plant Phenotyping (CVPPP) workshops	3.8	Various; see citations in review paper	Tsaftaris & Sharr 2019
Image analysis for automatic disease diagnosis in multiple crops using PlantVillage Nuru	3.8	Convolutional neural networks	Ramcharan et al. 2019

## 3. Data challenges

### 3.1 Data diversity and continuing obstacles to data sharing

Biological research tends to be very fragmented compared with other sciences, and biological data is highly heterogeneous as a result ( Hey et al. 2009; Marx 2013; Leonelli 2019; Strasser 2019). A key reason for this is the attention paid by biologists to the unique characteristics of the *target systems* that they are studying: different species of mushrooms, bacteria, trees, ferns and mammals can behave and interact with their environment in fundamentally different ways, which in turn affects their different structures, functioning and reproduction. Biodiversity thus encourages the production of research methods and instruments specifically tailored to the ‘endless forms most beautiful’ in question—with different laboratories producing data in a wide variety of ways. Added to this, there is the multiplicity of *purposes* for which biological research is conducted, which in the plant and crop sciences include the production of genetically engineered crops, understanding growth conditions, improving crop yield and identifying medically useful compounds; many of which also require the study of key environmental features such as soil and climate conditions. Moreover, the translation of plant research into agronomic spaces is made especially complex by the multiplicity of *stakeholders*, with breeders focused on the specific conditions in their target markets, farmers producing a large variety of data of potential research interest as part of their everyday work, and many companies working in agritech (including companies producing sensing devices for farms), although many data producers remain secretive around their own data practices and datasets. Furthermore, there is a *divergence* between the large emphasis on omics data within academic plant science and the equally strong focus on phenotypic data for crop evaluation favoured in more applied domains, which is only partly mitigated by ongoing efforts to bridge this gap and exploit the complementary nature of these data resources through integration and interoperability. Last but not least, there is *no consensus* on data formats, standards and methods of analysis. Datasets are typically collected with a specific hypothesis or practical use in mind, with much data not generated in machine-readable formats and data standards rarely prioritized when developing new methods or technologies. Data *circulation* is also limited, due to a lack of targeted incentives and necessary infrastructures as well as a general reluctance from researchers to share their data beyond their immediate communities of collaborators. Many research funders and institutions do not yet provide concrete incentives to make data publicly available, including rewards and resources to match the significant labor involved. This has significant implications for researchers, especially given the competitive culture predominant within the life sciences and the well-founded fear that spending resources on data curation may lower the publication rate of any one group, with negative effects on their reputation and future endeavors ( Leonelli et al. 2017; European Commission 2017).

This fragmented data landscape limits the opportunities for the application of AI to plant research and agronomy. For example, when object recognition software is applied to human faces, relatively homogeneous reference sets of photographs are available for training, but equivalent data is not available when the same technologies are aimed at identifying morphological traits in plants. The introduction of the FAIR principles ( Wilkinson et al. 2018), stating that data should be Findable, Accessible, Interoperable and Reusable, has greatly helped to address some of these issues. ^4^ Some organisations are promoting the “FAIRification” of data using semantic web technologies (e.g. https://www.go-fair.org), but even more limited forms of annotation, semantification and standardisation would significantly facilitate applications within more restricted domains. Many molecular biology data are already integrated in structured, curated and interlinked public repositories ( Rigden & Fernández 2020), which are widely used by the research community. This is not surprising given the historical ties between the development of sequencing technologies and the emergence of computation ( November 2012; Stevens 2013; Strasser 2019) and related database standards and classification initiatives ( Mackenzie et al. 2013) - often starting with data from model organisms grown in standard conditions like *Arabidopsis thaliana* with large associated research communities ( Leonelli & Ankeny 2012).

At the same time, many other types of data are not as standardised, and the heterogeneity of data formats and methods across different areas of the life sciences is likely to affect the ways in which FAIR principles are implemented. Such differential adoption of FAIR principles and resources may, again, constrain the potential for ML to integrate data across multiple domains. Indeed, while the FAIR data principles are increasingly being applied across the plant sciences ( Rodriguez-Iglesias et al. 2016; Pommier et al. 2019; Reiser et al. 2018), different projects have developed different elements of FAIR depending on their specific goals and context. Some applications, such as FAIDARE (FAIR Data-finder for Agronomic REsearch) ^5^ have focused on Findability. Others, such as the Crop Ontology and related ontologies in the Planteome project, have focused on interoperability and semantic standards. AI and ML applications depend heavily on the Interoperability and Reusability dimensions of FAIR, but these have received less attention overall than Findability and Accessibility. As well as the semantic efforts mentioned above, more recent initiatives such as BrAPI (Breeding API; Selby et al. 2019) and MIAPPE (Minimum Information about a Plant Phenotyping Experiment; Papoutsoglou et al. 2020) have addressed these aspects in a more targeted way.

Acknowledging and rewarding those who generate data would go a long way towards encouraging effective data sharing. One approach to this issue is exemplified by the Annotated Crop Image Database, which is set up to show only fragments of annotated images of plant phenotypes, without necessarily showing the detailed metadata that would allow others to re-use those images for biological research. ^6^ This encourages biologists to share their data as early as possible to support the development of methods such as feature detection, while at the same time protecting those data from re-use by other biologists for as long as it is needed for the original data producers to publish their own results. This is only one among many possible solutions to adequate acknowledgement of data sourcing, with other approaches favoring early data publication (for instance in data journals) as a way to reward data producers while also fast-tracking data sharing. The Research Data Alliance is one among many organizations engaged in developing conventions and methods to reassure those providing data that their own research and publications will not be adversely affected, such as for instance the CARE and the TRUST principles (Lin et al 2020). ^7^ It is imperative that such guidance is visibly implemented and that researchers are trained to understand its significance for their own work and data management strategies.

### 3.2 Selecting and digitising data

Given the wide variety of data types, formats and sources in the plant sciences, determining which data resources could be selected for AI-informed analysis constitutes a serious challenge. Are there data sets of immediate potential if suitably curated, and what metadata is needed to describe data sets so that their suitability for inclusion in a given analysis can be assessed? The achievement of clear criteria and priorities for data selection is a crucial issue given the considerable amount of work required to digitise, curate and process datasets and related metadata. Such criteria should consider the ML task at hand, the scientific goals as well as the concerns of individuals and groups holding the data.

Consider herbarium specimens as a promising potential substrate for ML. Collectively, the world’s herbaria contain an estimated 392,353,689 plant specimens as of December 2019 ( Thiers 2020), associated with metadata describing the place and time of their collection. ML can be used to infer useful information from the physical and molecular characteristics of the specimens to support automatic identification of plants ( Carranza-Rojas et al. 2017), or to find material with potentially useful traits ( Younis et al. 2018). Recent efforts have combined specimen images, their associated metadata including descriptive labelling, and associated field images ( Carranza-Rojas et al. 2017). These approaches could be used to monitor ex situ conservation efforts, to track changes in natural and farmed distribution of species in response to environmental changes, to trace the spread of invasive weeds, or many other applications not strictly related to crop research. However, many herbaria are only partially digitised, if at all. Most specimens have not been imaged or subject to molecular analysis, and even basic metadata is often not databased, but only exists in the form of hand-written or typed annotations attached to the physical specimen, meaning that even taking an inventory of stock is not possible, and access to the material is only possible via physical visit. Thus, while the new technologies of imaging, molecular analysis and ML have created new possibilities to exploit these historic collections ( Soltis 2017), these will remain unrealised until the information they contain is extracted, digitised, and made publicly available, tasks which are very labour-intensive. Interestingly, ML itself may be able to help solve this problem: the transcription of physical herbarium labels may be supported by the use of ML to interpret handwriting. A useful step towards this is the recent production of a benchmark dataset of transcribed herbarium labels ( Dillen et al. 2019), which could be used to assess the performance of algorithms. This does not however help to address questions of data selection. Researchers still need to decide which specimen and related data/metadata to prioritise given limited resources and the vast scale of existing collections. In turn, the selection of usable and relevant data and digitisation of records is tightly associated with the prioritization of research problems and questions on which to work. There is relatively little investment in improving procedures and methods in this area, and yet there is a need for processes through which researchers explicitly consider and debate which data should take precedence and why. Without such processes, the ensemble of data being curated risks being patchy and fragmentary, the random result of individual efforts by separate and uncoordinated projects rather than of a community effort to locate and invest on data of most relevance to all. Indeed, without such processes, pressure to use automatic methods, and to be seen using them, can aggravate the problem with researchers investing resources in the creation of large datasets without considering whether and how those data could be used.

### 3.3 Linking data to material samples

Clear reporting on the relation between digital data and material samples – the seeds, germplasm and other biological sources to which data are associated - is vital to the interpretation, re-use and reproducibility of results ( Leonelli 2016, Strasser 2019), as well as constituting a major source for data in the first place ( Bebber et al. 2012). Moreover, the use of plant science to inform agriculture and related domains such as forestry is predicated on understanding and utilizing the widest possible range of biological variation between and within species. For example, crop breeding is dependent on having access to a large pool of traits that can be incorporated in new varieties that are resistant to changing climates, diseases and stresses ( Hufford et al. 2019). Applications of AI to plant and related data must be designed in such a way that data, models and other outputs can be linked back to the material samples on which scientific research and biotechnological applications depend.

This has proved to be problematic. While a vast number of accessions of crops and crop wild relatives are held in genebanks worldwide, the corresponding data records for this global resource are often limited at both a scientific and operational level. Some progress has been made in promoting data deposition and thereby indexing of resources in overarching international plant genetic diversity databases such as EURISCO, which provides information about more than 2 million accessions of crop plants and their wild relatives, preserved *ex situ* by almost 400 institutes including both passport data and phenotypic data. ^8^ However, meeting the disparate needs of users, donors, funders and other stakeholders in such indexing databases remains difficult. Within the international phenotyping community, information systems are developing which require all objects, including individual plants, to be allocated a persistent URI ( Neveu et al. 2019). This increase in specificity has the potential to increase connectivity between phenomic data and the samples from which it was obtained, but comes with a significant overhead cost and to date is only feasible in indoor, highly mechanised environments.

Legacy systems do not always lend themselves to easy integration and can make consistent matching of appropriate terms and datatypes between originating resources difficult. There can be competing arguments for the most appropriate, efficient, or scientifically accurate representation or classification of data and characteristics to meet perceived audiences. A reluctance or inability to re-invent domain specific resource catalogues is also understandable given the range of operational concerns that inform the management of live resources. Genebank databases have been iteratively customised to user requirements and/or contractual constraints over a period of many decades. There may be significant conflicts between visibility and dissemination drivers for commercial and public collections and even for separately donated materials within those resources. There may also be concerns about third party use of collated data or perceived availability of materials, particularly where there may be implicit or implied intellectual property, or regulatory compliance benchmarks for benefit sharing obligations. A precautionary principle not to include portions of the biobank collection may also apply when downstream use of data or implied ownership of downstream discovered characteristics are considered by the biobank review panel considering inclusion in such an external index.

Within plant phenotyping facilities, the legacy problem arises from the historic variations in metadata collection. In particular, useful linkage of phenomic data to samples requires details of the growth environment to also be collected. This is now attracting significant interest, and methods and standards for e.g. illumination conditions are emerging ( Cabrera-Bosquet et al. 2016). Inclusion in some databases is now conditional on capture of specified levels of environmental information. Legacy data, however, often lack such information, and the variations in plant structure and performance introduced by environmental conditions - even within well-controlled environments - means that simple linking of genotype to phenotype is insufficient.

Between them, these issues can make reduce data donation to a lowest common denominator of permitted and approximated metadata overlap for a subset of holdings – often a simple index or indicator of materials which merely points to the originating collection and may not permit broader aggregation of recorded characteristics. This can be insufficient for useful exploitation of the resource by specialist researchers and will often render an aggregation site unpopular or secondary to the primary biobanks. The problem is even further exacerbated when considering the very large number of valuable land races, crop wild relatives (CWRs) and heritage varieties preserved *in-situ* at herbaria, botanical gardens and conservation sites. Moreover, and despite significant work invested in creating genotyping panels and populations for many different species, a lack of phenotypic data about accessions has limited the utilisation of this diversity and constrained understanding of the genetics of complex traits, leading to a phenomics “bottleneck” ( Araus & Cairns 2013). An increasing number of high-throughput phenotyping platforms are being constructed, in which large quantities of data about individual plants are collected, integrated and analysed with the help of ML techniques (especially on multispectral and RGB imaging data). These phenotyping platforms are at the forefront of materials-data linkage and biodiversity studies in plant science, and yet they are often unavailable beyond the institute or research group that developed them, for reasons ranging from data size to commercial protections.

The challenges of managing the relationship to material samples are not limited to datasets, but also include models. The accuracy of Genomic Selection (see Box 1) for a given breeding population is strongly dependent on genotypic and phenotypic data collected from closely related populations, which are used to train models ( Spindel & McCouch 2016). Robust linkage between models and the material samples for which they have been optimised, combined with pedigree data and made available via public infrastructures, will be important to enhance the accuracy and utility of GS modelling through greater transparency, comparison and reuse of models for related breeding materials or traits. Thus, the usefulness of AI-informed analysis of digital data is tied to investments in the development and maintenance of material samples - including those kept in seed banks and herbaria - and key germplasm metadata such as those captured by the Multi-Crop Passport Descriptors ( Alercia et al. 2015).

### 3.4 Standardising data and metadata

Standards ensure that data are collected in formats and with labels that can be understood by users, whether human or machine, as well as ensuring that a necessary minimum of contextual information (or metadata) is recorded about the methods through which data were generated and the environmental and experimental conditions in which they were acquired. Providing metadata labels and labelling/annotating individual data points with semantic standards both present major challenges for the use of ML and AI, although these challenges can differ in nature (the type and choice of standards required) and scale (labelling data points requires substantially more labour than assembling appropriate metadata). Nevertheless, many of the key issues of how to develop appropriate standards for labelling that fulfil the needs of different user communities and are widely adopted by those communities are shared between the two areas, and are increasingly approached through coordinated effort.

Consider this example of a dataset from orchard management. A two-year study of 19 orchards in New York state collected data on the effects of conventional pesticide use on the wild bee community visiting apple ( *Malus domestica*) within a gradient of percentage natural area in the landscape ( Park et al. 2015). ML techniques, such as hidden Markov models, can effectively be used to model the behaviour of pollinators based on movement data, especially between orchards and natural habitats. This in turn could inform decision support tools for scheduling the use of pesticides to limit their effect on pollinators, using data collected from individual trees by remote sensing technology. However, the dataset presents several issues for reuse. Each orchard was going to be visited twice for data collection, once before and once after blossoming, but the first year some data were not collected due to cancelled visits. While this was not a problem for this study, where the focus was on the bee count in the second year, for a study with a different objective the incomplete data could be problematic. Moreover, dates are annotated relative to bloom rather than to calendar dates; and a key variable is the Bee Impact Quotient (BIQ) for each individual pesticide, and other scores derived from these. These measures are appropriate for a study on pollinators, but may be less suitable for a study measuring different impacts on the ecosystem, such as plants or biodiversity. Without a preliminary discussion of standards for future data reuse at the start of the study, and incentives to ensure that the scientists involved are given credit for developing data resources of wider interest than for their own project, such considerations are not taken into account, and data collection cannot yield standardised, machine-readable labels for individual data points that can be aggregated and reused within other projects.

To counter such issues and help researchers to signpost more clearly the characteristics and expected utility of datasets, there has been considerable progress in developing semantic standards for data and metadata by a variety of transnational organisations and initiatives. For phenomic research, such efforts include the Ontologies community of practice of the CGIAR ( Arnaud et al. 2020), which manages the Crop Ontology, and the Food and Agriculture Organization’s AGROVOC thesaurus. Distinctive to both initiatives is not only the standardisation of terms for field studies, but also the attempt to develop terminologies that bridge the expertise of the multiple stakeholders in agricultural field trials, including farmers, breeders and scientists, and link different languages. ^9^ Initiatives such as ELIXIR, the Research Data Alliance Agricultural Data Interest Group, GODAN and the project PHENOME-EMPHASIS provide precious collaborative venues to improve plant data standards beyond molecular omics and experiments. Notable concrete examples include projects such as the Breeding API (BrAPI; Selby et al. 2019) and MIAPPE (Minimum Information about a Plant Phenotyping Experiment; Papoutsoglou et al. 2020), the latter fostered by ELIXIR as a way to improve consensus on ways to annotate data generated by phenotypic experiments; the Working Group on Integrating Genomic and Phenotypic Data for Crop and Forest Plants coordinated by ELIXIR-EXCELERATE ^10^; and the efforts to standardise the collection and interoperability of field data in the CGIAR’s AgroFIMS, the open-source FieldBook application ( Rife & Poland 2014) and the Grassroots information infrastructure of the BBSRC Designing Future Wheat programme. Efforts such as the COPO platform also implement semantic standards, including MIAPPE, in user interfaces to aid data brokering which underpins the availability of well-described datasets that can in turn power AI/ML studies ( Shaw et al., 2020).

These initiatives, and a shift in research culture more generally, are playing a central role in establishing wider attention to and use of best practice in standards to deliver impact through AI/ML (cf. Leonelli et al. 2017). Ensuring that these standards are not implemented retrospectively, but rather they are adopted before data are actually produced, remains a key challenge. In this respect, companies that develop scientific instruments and research software have a crucial role to play. This is particularly evident in the case of data generated by remote sensing technologies, where the most prominent standards concern the technical levels of imaging and data processing rather than data curation. For example, a recent study of the impact of oil palm plantation in Indonesia based on a range of sources of satellite imagery attempted to assess the impact that historical changes in land use had on greenhouse gas emissions ( van Beijma et al. 2018). An outcome of the study was that comparing the outputs from different remote sensing sources was severely compromised not because of any challenges of changes to satellite technology but rather because there was no consistency in the classification of land use between the different remote sensing campaigns.

### 3.5 Evaluating the quality of reference data

Developing reliable ML tools is dependent on having adequate reference data (also referred to as ground-truth or training data) for model validation. Obtaining or accessing reference data for complex field environments poses a distinct challenge, due to the scale of data collection required and the associated problem that the high value of such data means that it is frequently held behind restrictions on access or licensing agreements (see section 3.7). Purpose-built platforms such as Rothamsted Research’s North Wyke Farm Platform allow the monitoring and control of multiple agricultural and environmental variables, from plant growth through soil health and water flows, generating detailed, multi-scalar data ( Orr et al. 2016). Such facilities are expensive and few, however.

Given that the generation of new data specifically for the purpose of training and benchmarking can be expensive and time-consuming, re-using already published data for these purposes is desirable. Implementing good data and metadata standards can reduce the cost and time of reuse, but standardisation alone does not allow the creation of benchmark datasets on demand. The utility and accuracy of algorithms is dependent on the quality of the datasets used to train them. Without sufficiently broad and unbiased training sets, algorithms will not have wide general applicability. It is therefore necessary to address statistical aspects of data sets in addition to the data management and stewardship principles described in the previous section. Data quality benchmarking has played a central role for example in genomics, with projects such as the MAQC/SEQC ( SEQC/MAQC III Consortium 2014), the MicroArray/Sequencing Quality Control initiative by the FDA, but quality standards also depend on the potential implications of decisions taken based on the information contained in the data. For example, the evaluation of ecological risks associated with GM crops or pesticide use need to happen based on more robust data than those procured via fundamental research. While published data sets such as those in genomics repositories, citizen science platforms or ecological data banks typically have undergone some quality checks, these are tailored to the requirements of the original context of the data collection. Re-using data sets to develop an algorithm serving a changed purpose requires a fresh assessment of the suitability and quality of the data set. The following questions can provide guidance for finding out whether representativeness and resolution requirements are fit for the specific context and purpose of the algorithm that is being trained with the data.

1. Are the variables used by the algorithm (or sufficiently close surrogates) included in the data set? Are the measurement methods sufficiently accurate and precise for this purpose? Have they been taken on a sufficiently elementary unit rather than on an aggregated level only? If the data collection covers a time period, have the measurements been taken sufficiently frequently?
2. Are the records complete? If not, are records simply missing at random or are there any patterns in the absence that might skew the results obtained by the algorithm?
3. Is the sampling method used to collect the data subject to any selection biases that were negligible for the conclusions of the original study, but could impact the results or interpretation of the algorithm?
4. Where data has been gathered from human experts (e.g. in image annotation for phenotyping), has subjective bias been identified? Is the set of experts used sufficient to capture possibly conflicting views? Have the annotators understood and been provided with appropriate tools for the task?

One example to illustrate quality issues in data reuse concerns the British Farm Scale Evaluations (FSE), which analysed the effect of genetically modified herbicide-tolerant varieties of beet, oilseed rape and maize and that of comparable conventional varieties on the abundance and diversity of arable plants and invertebrates ( Firbank et al. 2003). ^11^ The data set consists of complex time courses reliant on farmers’ assessments. While measurement of weed cover, crop cover, crop height and pollinators followed protocols, the schedule for taking the measurements throughout the year was chosen by the individual land managers, which made comparisons difficult. It was pointed out that: extra data assessing "whether there is evidence of biodiversity harm from the use of the GM crop and herbicide regime" should have been collected ( Environmental Audit Committee 2004); no definitive yield component had been included, which makes it difficult to use this data set for trade-offs between environmental and economic targets; and pesticide data is given as product application rates, which makes interpretation of these numbers difficult for future studies. Another major issue is missing data due to vandalism, a foot and mouth disease outbreak and unknown reasons, in some cases showing systematic patterns of incompleteness.

Indeed, a related issue is the breadth of data used to train models: whether they sufficiently represent the variation and diversity of target species or populations. Use of computer-generated images of plants in order to enlarge the image datasets used to train deep learning computer vision algorithms for phenotyping is increasingly common (e.g. Ubbens et al. 2018; Humphreys et al. 2018; Toda et al. 2020; Atanbori et al. 2020). Whether or not such methods could feasibly be used to generate training data that sufficiently reflected the complexity of field environments is another question. The expansion of field phenotyping, including attempts to capture, integrate and analyse imaging captured by drones and other sensing technologies, is likely to be necessary for this task. Progress in the latter area is rapid, although it is still constrained by the expensive and technically challenging nature of both experiments and associated data annotation practices ( Fahlgren et al. 2015; Coppens et al. 2017; Rosenqvist et al. 2019).

### 3.6 Using software and models across scales, species and environments

When developing effective AI solutions in plant-related research, access to adequate software and modelling platforms is as necessary as access to high-quality data. Software and models need to be implemented on digital environments that its users have access to or are willing to pay for. Accessibility, especially for large-scale AI, is key: researchers need access to the computing and data platforms needed to power AI at a reasonable price, in order for such research to be scalable. Where possible, software should also be portable for use across digital environments, so as to accommodate researchers working in different systems; and it should be approachable by users with a range of experience in handling and analysing data.

A key obstacle here is the fact that researchers who can formulate the biological problems are often not those developing ML algorithms. An example is the use of targeted software to explore existing data in search for new targets for experimental investigation. The KnetMiner resource for instance assembles a suite of software and data integration methods aimed at sifting through the biological literature and public data resources to explore relationships between datasets and species, especially in cases where multiple traits are connected to multiple genes. The application of these exploratory methods to key crops such as wheat, sorghum and sugarcane has already resulted in the identification and further study of important agronomic traits ( Hassani-Pak et al. 2020). At the same time, it requires expert tailoring whenever targeting a new species, including biologically informed assessment of which datasets used for key crops can be applied to other crops. Indeed, not all models will work directly off the shelf on a new dataset/problem. Giuffrida et al. (2019) devised an algorithm that adapts a leaf counting model on new data without requiring annotations and without requiring the availability of the original training dataset. As model complexity increases, however, so does the opacity of the models. Dobrescu et al. (2019) sought to develop mechanisms that help peek into what ML models learn in tasks of object counting (and in particular leaves) – part of a growing research field which promises to make CNN methods better understood, increasing trust in the insights they provide.

It is then not enough to provide researchers with software alone. Rather, this must be supported with workflows that incorporate the whole life cycle of data preparation, validation and analysis, and which can be operationalised with minimal friction ( Bechhofer et al. 2010). Algorithms and data models must also be articulated at the right level of abstraction, to resolve what could be perceived as a new form of ‘translation gap’ between the cutting edge of data science and the frontiers of plant research. Software and models created for very particular uses will have higher requirements for data quality and annotation, creating a barrier to reuse ( Tiwari et al. 2020; Stanford et al. 2015). Some models need to be flexible enough to work across the multiple scales that characterise both biological work in general and agricultural research in particular, including between species and between different environments. An example is the John Innes Centre’s work to create data resources with the appropriate software that enable the transfer of learning from model organisms (e.g. *Arabidopsis*) to non-model organisms (e.g. *Brassica* crops) ( Jones et al. 2018, 2020; Calderwood et al. 2020a, 2020b). ^12^ Many crops have large complex polyploid genomes, one of many factors that can make the direct transfer of knowledge problematic. Machine learning approaches are being developed that allow for large transcriptomic and phenotypic datasets being collected from many individuals, populations and species. This in turn can be exploited to identify similarities and differences in the regulation of developmental transitions in response to environmental stimuli ( Calderwood et al. 2020a, 2020b). Bringing foundational plant science into the crop space is crucial, yet key challenges remain at every level from gene activity and function through networks, tissue behaviour and plant physiology to field-level behaviour.

The need to operate across multiple scales has long been acknowledged to require trade-offs between accuracy and generality ( Levins 1966). Indeed, most models are designed to address specific questions and will not be applicable across scales. This raises questions around whether and how the results of such modelling efforts can be linked and integrated. The goal of ML is not to ensure how the model will do on the training data but instead how it will perform on a testing set. The testing set is used to ascertain how well the model will generalise to an unseen data source and thus “in the wild”. After all, we do care about creating AI/ML that will generalise well either in unseen data or unseen tasks. For example, how will a model that is trained to count plant leaves perform when tasked to count leaves in different images of the same plant family (different illumination), or a different plant family or even a different task (e.g. seed counting)? The ability for models to generalise is largely governed by the quality of the internal data representations the model has learned to fulfil the task. If one relies on supervised machine learning, then these representations will be tuned to the specific task and will have difficulty in generalising. Here multi-task and meta-learning can help as they tend to learn representations that can more easily generalise ( Dobrescu et al. 2020).

However, if we rely on annotations to drive this supervised learning of data representations, one must readily ask whether data quality and annotation play a key role. In ML, data cleaning and preparation take a considerable amount of time. Even more time consuming is annotation/labelling of individual data points. Approaches to relieve the data annotation effort include semi-supervised, self-supervised and multi-task learning. These methods aim to learn representations by leveraging unlabelled data, or correlations and self-similarity of the data themselves, or correlations between tasks. Considerable and notable improvements have been made outside and within plant sciences, and particularly in image-based phenotyping. Yet even these methods rely on some annotations. Thus, one must consider whether noise (errors) in annotation have an effect. Learning with label noise, as it is colloquially known in ML, is a mathematical framework that aims to learn a good model even when labels may be noisy, i.e. have errors ( Natarajan et al. 2013). Recently, Giuffrida et al. (2018) went to the extreme of assessing such levels of noise amongst expert and even novice annotators (citizen scientists). The findings are promising: Despite the presence of noise, as long as multiple annotations of the same datum by diverse individuals exist, models can be learned.

One must consider errors in annotation not only in providing data and metadata labels for the datasets to which ML is applied, but also in how ML outputs will be used to support statistical hypotheses. In this aspect, an error in labelling the metadata of a mutant as control will create considerable propagation of error in the pipeline. Thus consistent records of experimental conditions will help ensure that such errors are minimised. Here ML can also help identify errors ( Schramowski et al. 2020). An ML algorithm can actually act as a calibration method: outputs of an ML model which are suddenly inconsistent point to data inputs that are out of distribution. Whether such out of distribution data are due to errors in the data or metadata or because the ML is encountering data not trained with (but could be updated), necessitates human intervention and this in turn creates a viable checkpoint in the development of robust data processing pipelines.

### 3.7 Managing data access responsibly

Access to appropriate datasets is necessary for the application of AI tools to complex environmental and biological research topics, yet it clearly depends on factors well beyond scientific need, including intellectual property regimes, data governance by specific institutions, and consideration of the rights and risks involved in data sharing for those who produce the materials from which data are extracted and/or may suffer the social and economic consequences of specific applications of data analysis ( Williamson & Leonelli 2022). Legal constraints such as intellectual property controls and licensing regimes can and often do put the data beyond the financial means of lower-resourced researchers and institutions, or place restrictions on the use of the data that makes the kind of wide-ranging data mining required for AI application difficult if not impossible to implement ( Jefferson et al. 2015). Given the distinctive landscape of intellectual property rights, contracts and the need to find incentives for data sharing that respond to imperatives of commercial competition, finding ways to make data usable to a range of actors without necessarily sharing it is likely to become increasingly important. In biomedicine, initiatives such as DataSHIELD have been developed in which users are able to run analyses on a dataset via an intermediary platform without having direct access to the source data ( Murtagh et al. 2012). Such efforts allow the anonymisation of data and removal of patient/volunteer personal information, which are recognised as important issues in biomedical research. Similar initiatives such as the Open Algorithms (OPAL) project, developed in relation to commercially sensitive data ( Roca & Letouze 2016), have recently been promoted in agricultural research forums such as the CGIAR Big Data in Agriculture Convention, but their uptake remains to be determined.

Research institutions including universities have often kept data from widespread access, with even data produced by publicly funded studies remaining either unknown or inaccessible to other researchers. This is partly explained by lack of investment in the platforms, curation expertise and training required to ensure data sharing and facilitate analysis, and partly due to enduring confusion around legal accountability of research institutions vis-a-vis the requirements of governments, data protection laws, private sponsors (including public-private-partnerships) and public funders - not to speak of the fact that researchers often operate within international networks where different national legislation and expectations may apply.

Data access must also be balanced against ethical concerns that have recently arisen around the re-use of data and materials collected in low-income countries and/or low-resourced research environments. With reference to the longer history of colonial exploitation of indigenous agricultural knowledge to support market-driven growth in the Global North (e.g. Carney 2001), international institutions including the World Data Systems, CODATA and the CGIAR have pointed to the potential for indiscriminate data access to accelerate so-called “digital feudalism”; the exploitation of more vulnerable members of the agricultural research network by better-resourced and more powerful actors (such as Alphabet/Google) who can effectively appropriate such data. The opportunities afforded by AI, while holding the potential to benefit many stakeholders, also create new commercial incentives for such exploitation.

Key areas for negotiation include access and benefit sharing agreements and the protection of sensitive data, for example where they include location or certain kinds of farm production data. In the biomedical field, strong regimes of governance and ethics have been developed for data protection and legislating the acceptable uses of data ( Hilgartner 2017), and these may provide a model for the plant sciences. However, plant data poses several different challenges to human biomedical data, notably the fact that much of the data utilised in basic and translational plant research does not come under the more protected category of personal data, but is frequently covered instead by contract law ( Wiseman et al. 2018).

### 3.8 Engaging experts beyond one’s domain

Despite the increased use of ML expertise and tools and the example set by some highly visible projects, collaboration between cutting-edge data science research groups and plant science communities is not yet commonplace ( Henkhaus et al. 2020; Department of Energy 2020). On the one hand, this is due to the poor visibility of plant science datasets and problems to the data science community, in comparison to more prominent biomedical or environmental data and challenges. On the other hand, plant researchers need a better understanding of how algorithms work and what can legitimately be expected from the outputs of AI and ML. It is necessary to up-skill researchers with expertise about the available types and minimum necessary semantic annotations that datasets must be labelled with in order to make them machine-readable, in the first instance, and usable with specific algorithms. Providing researchers in the plant sciences with a minimum fundamental knowledge about such matters, preferably from an early stage in their careers, will facilitate the deployment of AI in the field and assisting decision-making around the issues of data selection and management described above, while also acting as an incentive towards the implementations of standards in the production and use of plant data.

One example of combining community-wide incentives with collaboration and upskilling are the “data challenges” organised in conjunction with the Computer Vision Problems in Plant Phenotyping (CVPPP) workshops, held at various international computer vision conferences since 2014. The first challenges were built around a curated dataset of images of rosette plants, including *Arabidopsis* and tobacco, taken in a controlled experimental setting, that could be used to test algorithms for leaf detection, segmentation and counting. This dataset, provided with expert annotation and full metadata, was presented alongside clear problem statements for computer vision researchers to work with and scripts for preprocessing and to code performance metrics, thereby minimising the costs of engagement. Phenotyping problems were mapped onto appropriate computer vision terminology, for example leaf segmentation to multi-instance segmentation, and the workshops were organised to facilitate research likely to lead to publications for participants. These efforts resulted in wider visibility of the *Arabidopsis* dataset among the CV community as an important benchmark in the development of multi-instance segmentation and object counting tools ( Tsaftaris & Sharr 2019); educated ML researchers in the potential of plant data; and highlighted the potential of computer vision (and AI tools more generally) in addressing long-standing plant research questions. ^13^ At the same time, this example highlights the significant effort involved in developing closer collaboration between these two research communities, since presenting the dataset required extensive preparation by the organisers (who needed an understanding of both areas of work to effectively set up the challenge). In addition to supporting access and use of specific software, hardware and workflows, there are benefits to be gained from supporting engagement with tools around which a community has developed, particularly when the users may lack technical background in ML/software engineering. Access to other users’ experiences and opinions is likely to be very valuable here, whether it is informal or through training material and events.

It is crucial to extend this engagement beyond the sphere of professional scientists to include other stakeholders in food systems, including farmers, agronomy advisors, plant breeders, food manufacturers and suppliers, nutritionists and others. Without dialogue with and among stakeholders, it is hard to identify the priority areas — the social-scientific needs and challenges — where there is greatest opportunity for AI applications to achieve impact. Mapping the stakeholder networks for specific forms of data-intensive plant research is a labour-intensive but important endeavour ( The Open Research Data Taskforce 2018), as demonstrated in large projects such as Elixir that devoted significant efforts towards developing transparent and robust mapping services. Government representatives, funding agencies and industrial partners need to be engaged in the development of any data infrastructures and services. The involvement of industrial partners in particular is crucial given their ownership of key data resources, and also for their use of the tools and applications of their outputs. There is strong need for increased governance and related norms ensuring the delivery of public goods from those organisations that see data as a key part of their commercial activities - similarly to what the Food and Agriculture Organisation has been spearheading in the case of plant genetic resources. If the field is to provide advantages to a wider range of socio-economic actors, SMEs also need to be represented in future discussions and governance strategies around data access and protections. In developing its Agri-tech strategy, ^14^ the UK government identified the key role of data and placed the development of an Agri-tech centre dedicated to data integration and access (Agrimetrics) ^15^ as central to its wider development of centres of agricultural innovation. ^16^ Such collaboration has also been envisioned, for instance, in the work of the Agrisemantics working group within the Agricultural Data Interest Group of the RDA ^17^ and the CGIAR Communities of Practice bringing together stakeholders to discuss data standards and semantics ( Arnaud et al. 2020). This engagement is crucial to ensure that academic expertise is informing and contributing to food security on the ground. Equally important is for public academic research, typically targeted to a wider range of topics, crops and applications, to be directed towards stakeholder needs. For instance, PlantVillage Nuru, a free smartphone app that uses automated image analysis and recognition with a phone camera for immediate disease diagnosis in several other crop species ( Ramcharan et al. 2019), is targeted at farmers in the developing world and was specifically designed, in consultation with farmers’ representatives, to be usable offline and with minimal external input. This resulted in wide uptake and positive feedback due to the accessibility of the app to farmers and the usefulness of its contents and design.

## 4. Conclusion: what data landscape do we need for plant-related AI?

We have reviewed eight data challenges that need to be urgently confronted in order to support the application of AI and ML tools to plant-related research (see Table 1). With specific reference to the UK and Europe, where our work is based, we discussed examples of good practice, including efforts to articulate data standards, algorithms and models at the right level of abstraction, in order to fit existing research questions and also address the gaps separating cutting edge data science from the frontiers of plant research. Building on such examples, we pointed to the need for a more systemic change in how research in this domain is conducted, incentivised, supported and regulated. We highlighted the importance of developing data services aiming to make data available and usable to people. This is particularly important in relation to environmental data of relevance to plant research, on which there has been much less focus compared to the tools already present to cope with genomic data. We pointed to the need for substantive investment in the development and maintenance of data infrastructures, standards and software, as well as: venues and training programmes aimed at fostering collaboration among the diverse expertise required (and especially exposure to data science for plant scientists and breeders); the identification of relevant stakeholders including industry, governmental agencies, local breeders and indigenous communities as relevant; and substantive engagement with those stakeholders. We stressed the difficulties in implementing these approaches within the highly fragmented biological data landscape, and the even more complex ensemble of public and private sponsors involved in research on crops. Despite marked advances in data availability, infrastructures and analytics, many plant researchers remain unaware of the extent to which AI tools could support their work, and do not actively participate in the effort to produce reliable data for the community.

One way to shift incentives and support a substantive culture change among researchers could be to foster international and transdisciplinary collaboration around big projects with clear use cases - a “moonshot” equivalent to the Human Genome Project or the search for the Higgs particle in physics. Big science of this kind has a strong track record in driving the development of standards and epistemic cultures, as well as bringing together international partners to maximize the strengths of different regions and approaches ( Leonelli & Ankeny 2015). The agronomic domain may need one such big project to create traction and new forms of collaboration, especially given the importance of driving adoption of common standards across as diverse research communities as those of data scientists working on algorithms, molecular biologists focused on genetic engineering and crop scientists engaged in field experiments. Targets for such a moonshot project could be: addressing the phosphate crisis and its impact on agricultural yield; developing a fully digital farm modelling an existing experimental station; or the development of ecosystem services using multiple metrics.

An alternative approach would be to focus on a key feature of ML that has been lacking in previously dominant technologies: its ability to both generalise and transfer between domains, once specific, targeted solutions are found to well-defined problems. Once a machine learning strategy has been identified for a given task exposure to further examples of that task typically improves performance, sometimes even when the details and environment are significantly different. Rather than identify a moonshot biological challenge, which runs the risk of creating more tools tuned to specific research questions, an explicit search for capabilities needed across a range of plant and agricultural science scenarios could inform the identification of Technological Grand Challenges facing this community. These could be used both to spread innovation across the community and to engage colleagues from other disciplines. This approach could learn from other areas of research who have fared better in the development and application of AI, such as biomedicine. Repurposing some of the insights and infrastructures created in that domain would also be very useful for plant-focused science, including in tackling ethical and governance issues associated with the protection, sharing and reuse of plant data.

Any future strategy for the development and application of AI in plant-focused research will need to have data curation at its centre, rather than as an afterthought. Making plant data FAIR is crucial. This in turn requires *both* technical work on standards, reference data, software and modelling, *and* organisational work towards establishing norms and venues for appropriate data governance (including on the terms of ownership, access to and reuse of data) as well as engagement with the widest possible spectrum of relevant stakeholders. Most importantly, it requires collaboration towards tailoring the technologies to the challenges posed by the green domain and the role of plants in relation to food systems and environmental sustainability. The opportunities immediately available in terms of AI applications may not necessarily be what plant research and agronomy need. There is a need to foster collaboration between fundamental researchers, data scientists, algorithm developers and end users in order to identify and maximise opportunities in this domain. Notably, while overcoming challenges to the effective use of AI will require changing practices and networks, it is important that such changes should not detrimentally affect what has already been successful. Existing communities of practice (such as the ELIXIR plant science community in Europe and the RDA agriculture-related groups at the global level) provide valuable sources of expertise and collaboration, and disrupting these risks creating more obstacles to good practice than benefits. We should note that making data FAIR along these lines will not resolve all issues of comparability and interoperability across experiments, given the enormous variability in settings and the number of variables involved - all of which are regularly updated to reflect local conditions. Carrying out a meta-analysis of data across experiments using AI will thus always require calibration and adjustments to allow for the specific sites, purposes and conditions of the study.

Last but not least, improvements in data management may help identify and account for ethical and societal issues of relevance to agronomy and food production. There has been widespread concern that the adoption of ML tools implies a decrease in the oversight and control retained by humans on the interpretation of results, including the assessment of the potential implications of any resulting actions for stakeholder communities such as farmers, breeders and consumers. This has been flanked by worry around documenting the provenance of data and rewarding the efforts involved in generating the materials and conditions for data collection, especially where results are extracted from farming communities in deprived areas. Practical solutions to these concerns require concerted effort from data producers and curators, research institutions, data infrastructures and international governance (see also Williamson & Leonelli 2022). For instance, the impact of specific crop varieties on diverse landscapes is considered by AgroFIMS and other tools developed by the CGIAR, while the allocation of ownership claims and rewards attached to discovery is incorporated into the Global Information System (GLIS) of the International Treaty on Plant Genetic Resources for Food and Agriculture. Thus, data management strategies can help to ensure that the environmental, social and economic impact of AI tools is built into all applications.

## Data availability

No data are associated with this article.

## Footnotes

^1^

https://www.marketreportsworld.com/global-artificial-intelligence-ai-in-agriculture-market-13268433.

^2^
We recognise that there is disagreement over whether ML can always be classified as AI, given that the application of ML techniques often requires extensive manual feature extraction in order to process data more effectively for analysis. In this regard, ML may be considered closer to statistical methods than to AI. For the purposes of this paper, where many of the data challenges are shared between existing methods of ML and AI *sensu stricto*, we will treat the two as a continuum of techniques where AI is the more encompassing and general term.

^3^
A list of examples of ML and AI applications discussed in this paper is provided in Table 2 at the end of this article.

^4^
In short, the existence of the data should be published, procedures for accessing the data should be available, sufficient metadata should be provided to allow the data to be understood and appropriately repurposed and common formats and APIs should be used to facilitate the integration of different data sets.

^5^

https://urgi.versailles.inrae.fr/faidare/.

^6^

https://plantimages.nottingham.ac.uk/.

^7^

https://www.gida-global.org/care#.

^8^

https://eurisco.ipk-gatersleben.de/.

^9^
Semantic standards that recognise and incorporate this diversity of knowledge will be a necessary bedrock for any applications of AI and ML that are envisioned to work for diverse user bases, and to preventing implicit bias towards the terminology, scope or aims of dominant research groups ( Arnaud et al. 2020).

^10^

https://elixir-europe.org/about-us/how-funded/eu-projects/excelerate/wp7.

^11^

The datasets are published by CEH as a collection here https://catalogue.ceh.ac.uk/documents/876358e4-62f7-4386-99e1-7d3eac223e03. Each crop dataset has its own DOI and the metadata gives a summary of measurements/data available, plus an extra dataset for management data.

^12^

http://order.jic.ac.uk.

^13^
Another successful initiative by the CVPPP is the Global Wheat Detection Kaggle Competition launched to broaden engagement in summer 2020, which received over 2000 entries ( https://www.kaggle.com/c/global-wheat-detection).

^14^

https://www.gov.uk/government/collections/agricultural-technologies-agri-tech-strategy.

^15^

https://agrimetrics.co.uk/.

^16^

https://www.agritechcentres.com/.

^17^
See e.g. https://www.rd-alliance.org/system/files/documents/SEMANTICS-RICE_poster_LD.jpg.

## References

[ref1] Addy JWG Ellis RH MacDonald AJ : Investigating the effects of inter-annual weather variation (1968-2016) on the functional response of cereal grain yield to applied nitrogen, using data from the Rothamsted Long-Term Experiments. *Agric For Meteorol.* 2020;284(15):107898. 10.1016/j.agrformet.2019.107898 32308247 PMC7079297

[ref2] Alercia A Diulgheroff S Mackay M : *FAO/Bioversity multi-crop passport descriptors V.2.1.* 2015; Rome: Food and Agriculture Organization of the United Nations & Bioversity International. Reference Source

[ref3] Araus JL Cairns JE : Field high-throughput phenotyping: The new crop breeding frontier. *Trends Plant Sci.* 2013;19(1):52–61. 10.1016/j.tplants.2013.09.008 24139902

[ref4] Arnaud E Laporte M-A Kim S : The Ontologies Community of Practice: A CGIAR Initiative for Big Data in Agrifood Systems. *Patterns.* 2020. 10.1016/j.patter.2020.100105 33205138 PMC7660444

[ref5] Atanbori J French AP Pridmore TP : Towards infield, live plant phenotyping using a reduced-parameter CNN. *Mach Vis Appl.* 2020;31:2. 10.1007/s00138-019-01051-7 31894176 PMC6917635

[ref6] Bebber DP Carine MA Davidse G : Big Hitting Collectors Make Massive and Disproportionate Contribution to the Discovery of Plant Species. *Proc Biol Sci.* 2012;279(1736):2269–74. 10.1098/rspb.2011.2439 22298844 PMC3321708

[ref7] Bechhofer S Roure DD Gamble M : Research Objects: Towards Exchange and Reuse of Digital Knowledge. *Nat Preced.* 2010. 10.1038/npre.2010.4626.1

[ref8] Birney E Clamp M Durbin R : GeneWise and Genomewise. *Genome Res.* 2004;14:998–995. 10.1101/gr.1865504 15123596 PMC479130

[ref9] Cabrera-Bosquet L Fournier C Brichet N : High-throughput estimation of incident light, light interception and radiation-use efficiency of thousands of plants in a phenotyping platform. *New Phytol.* 2016;212:269–281. 10.1111/nph.14027 27258481

[ref10] Calderwood A Hepworth J Woodhouse S : Comparative transcriptomics identifies differences in the regulation of the floral transition between Arabidopsis and *Brassica rapa* cultivars. *bioRxiv.* 2020a;2020.08.26.266494. 10.1101/2020.08.26.266494

[ref11] Calderwood A Lloyd A Hepworth J : Total FLC transcript dynamics from divergent paralogue expression explains flowering diversity in B. napus. *New Phytol.* 2020b. Accepted. 10.1111/nph.17131 PMC7986421 33289112

[ref12] Carbonell IM : The ethics of big data in big agriculture. *Internet Policy Review.* 2016;5(1). 10.14763/2016.1.405

[ref13] Carney JA : *Black Rice: The African Origins of Rice Cultivation in the Americas.* Cambridge, MA: Harvard University Press;2001.

[ref14] Carranza-Rojas J Goeau H Bonnet P : Going deeper in the automated identification of Herbarium specimens. *BMC Evol Biol.* 2017;17:181. 10.1186/s12862-017-1014-z 28797242 PMC5553807

[ref15] Coppens F Wuyts N Inzé D : Unlocking the Potential of Plant Phenotyping Data through Integration and Data-Driven Approaches. *Curr Opin Syst Biol.* 2017;4:58–63. 10.1016/j.coisb.2017.07.002 32923745 PMC7477990

[ref16] Crossa J Pérez-Rodríguez P Cuevas J : Genomic selection in plant breeding: methods, models, and perspectives. *Trends Plant Sci.* 2017;22(11):961–975. 10.1016/j.tplants.2017.08.011 28965742

[ref17] Data Study Group Team: Data Study Group Network Final Report: Rothamsted Research. *Zenodo.* 2020, April 29. 10.5281/zenodo.3775489

[ref18] Department of Energy: *AI for Science Report.* 2020. Reference Source

[ref19] Dillen M Groom Q Chagnoux S : A benchmark dataset of herbarium specimen images with label data. *Biodivers Data J.* 2019;7:e31817. 10.3897/BDJ.7.e31817 30833825 PMC6396854

[ref20] Dobrescu A Giuffrida MV Tsaftaris SA : Leveraging multiple datasets for deep leaf counting. *Proceedings of the Computer Vision Problems in Plant Phenotyping (CVPPP), An ICCV workshop.* Oct 2017.

[ref21] Dobrescu A Giuffrida MV Tsaftaris SA : Understanding Deep Neural Networks for Regression in Leaf Counting. *Proceedings of the IEEE/CVF Conference on Computer Vision and Pattern Recognition (CVPR) Workshops.* 2019. Reference Source

[ref22] Dobrescu A Giuffrida MV Tsaftaris SA : Doing More With Less: A Multitask Deep Learning Approach in Plant Phenotyping. *Front Plant Sci.* 2020; (28 February). 10.3389/fpls.2020.00141 32256503 PMC7093010

[ref23] El-Gebali S Mistry J Bateman A : The Pfam protein families database in 2019. *Nucleic Acids Res.* 2019;47(D1):D427–D432. 10.1093/nar/gky995 30357350 PMC6324024

[ref24] Environmental Audit Committee: GM Foods—Evaluating the Farm Scale Trials. *Second Report of Session* United Kingdom House of Commons;2004;2003-04, Vol. I. Reference Source

[ref25] European Commission: Incentives and Rewards to Engage in Open Science Activities. *Thematic Report No 3 for the Mutual Learning Exercise Open Science: Altmetrics and Rewards of the European Commission.* 2017. Reference Source

[ref26] Fahlgren N Gehan MA Baxter I : Lights, camera, action: High-throughput plant phenotyping is ready for a close-up. *Curr Opin Plant Biol.* 2015;24:93–99. 10.1016/j.pbi.2015.02.006 25733069

[ref27] Firbank LG Heard MS Woiwod IP : An introduction to the Farm-Scale Evaluations of genetically modified herbicide-tolerant crops. *J Appl Ecol.* 2003;2–16. 10.1046/j.1365-2664.2003.00787.x PMC1693276 14561314

[ref28] Fu P Meacham-Hensold K Guan K : Hyperspatial leaf reflectance as a proxy for photosynthetic capacities: An ensemble approach based on multiple machine learning algorithms. *Front Plant Sci.* 2019;10:730. 10.3389/fpls.2019.00730 31214235 PMC6556518

[ref29] Gao J French AP Pound MP : Deep convolutional neural networks for image-based *Convolvulus sepium* detection in sugar beet fields. *Plant Methods.* 2020;16:29. 10.1186/s13007-020-00570-z 32165909 PMC7059384

[ref30] Giuffrida MV Minervini M Tsaftaris SA : Learning to Count Leaves in Rosette Plants. In: Tsaftaris SA Scharr H Pridmore T , eds. *Proceedings of the Computer Vision Problems in Plant Phenotyping (CVPPP).* BMVA Press;2015.

[ref31] Giuffrida MV Chen F Scharr H : Citizen crowds and experts: observer variability in image-based plant phenotyping. *Plant Methods.* 2018;14(12). 10.1186/s13007-018-0278-7 29449872 PMC5806457

[ref32] Giuffrida MV Dobrescu A Doerner P : Leaf Counting Without Annotations Using Adversarial Unsupervised Domain Adaptation. *Proceedings of the IEEE/CVF Conference on Computer Vision and Pattern Recognition (CVPR) Workshops.* 2019. Reference Source

[ref33] Gonzalez-Camacho JM Ornella L Perez-Rodriguez P : Applications of Machine Learning Methods to Genomic Selection in Wheat Breeding for Rust Resistance. *Plant Genome.* 2018;11(2):1–15. 10.3835/plantgenome2017.11.0104 30025028 PMC12962436

[ref34] Hayes WS Borodowski M : How to Interpret an Anonymous Bacterial Genome: Machine Learning Approach to Genome Identification. *Genome Res.* 1998;8:1154–1171. 10.1101/gr.8.11.1154 9847079

[ref35] Harfouche AL Jacobson DA Kainer D : Accelerating climate resilient plant breeding by applying next-generation artificial intelligence. *Trends Biotechnol.* 2019;37(11):1217–1235. 10.1016/j.tibtech.2019.05.007 31235329

[ref36] Hassani-Pak K Singh A Brandizi M : KnetMiner: a comprehensive approach for supporting evidence-based gene discovery and complex trait analysis across species. *bioRxiv.* 2020:2020.04.02.017004. 10.1101/2020.04.02.017004 PMC8384599 33750020

[ref37] Henkhaus N Bartlett M Gang D : Plant science decadal vision 2020–2030: Reimagining the potential of plants for a healthy and sustainable future. *Plant Direct.* 2020;4:1–24. 10.1002/pld3.252 32904806 PMC7459197

[ref38] Hey T Tansley S Tolle K : *The Fourth Revolution: Data-Intensive Scientific Discovery.* Redmond, WA: Microsoft Research;2009.

[ref39] Hilgartner S : *Reordering Life: Knowledge and Control in the Genomics Revolution.* Cambridge, MA: MIT Press;2017.

[ref40] Hufford MB Miery B Teran JC : Crop Biodiversity: An Unfinished Magnum Opus of Nature. *Annu Rev Plant Biol.* 2019;70:727–751. 10.1146/annurev-arplant-042817-040240 31035827

[ref41] Humphreys MW Doonan JH Boyle R : Root imaging showing comparisons in root distribution and ontogeny in novel Festulolium populations and closely related perennial ryegrass varieties. *Food Energy Secur.* 2018;7(4):e00145. 10.1002/fes3.145 30774947 PMC6360931

[ref42] Jefferson O Köllhofer D Ehrich TH : The ownership question of plant gene and genome intellectual properties. *Nat Biotechnol.* 2015;33(11):1138–1143. 10.1038/nbt.3393 26544141

[ref43] Jensen JL Schjonning P Watts CW : Soil degradation and recovery – Changes in organic matter fractions and structural stability. *Geoderma.* 2020;364:114181. 10.1016/j.geoderma.2020.114181 32255839 PMC7043339

[ref44] Jiang Y Li C : Convolutional Neural Networks for Image-Based High-Throughput Plant Phenotyping: A Review. *Plant Phenomics.* 2020;2020:4152816. 10.34133/2020/4152816 33313554 PMC7706326

[ref45] Jones DM Wells R Pullen N : Spatio-temporal expression dynamics differ between homologues of flowering time genes in the allopolyploid *Brassica napus.* *Plant J.* 2018;96:103–118. 10.1111/tpj.14020 29989238 PMC6175450

[ref46] Jones DM Olsson TSG Pullen N : The oilseed rape developmental expression resource: a resource for the investigation of gene expression dynamics during the floral transition in oilseed rape. *BMC Plant Biol.* 2020;20:344. 10.1186/s12870-020-02509-x 32693783 PMC7374918

[ref47] King B : Inaugural Address. *CGIAR Big Data Convention.* 2020;2020. Reference Source

[ref48] Larrañaga P Calvo B Santana R : Machine learning in bioinformatics. *Briefings in Bioinformatics.* 2006;7(1):86–112. 10.1093/bib/bbk007 16761367

[ref49] Leonelli S : What Difference Does Quantity Make? On the Epistemology of Big Data in Biology. *Big Data Soc.* 2014;1:1–11. Reference Source. 10.1177/2053951714534395 25729586 PMC4340542

[ref50] Leonelli S : *Data-Centric Biology: A Philosophical Study.* Chicago, IL: University of Chicago Press;2016.

[ref51] Leonelli S : The challenges of big data biology. *eLife.* 2019;8:e47381. 10.7554/eLife.47381 30950793 PMC6450665

[ref52] Leonelli S Ankeny RA : Re-thinking organisms: The impact of databases on model organism biology. *Stud Hist Philos Biol Biomed Sci.* 2012;43(1):29–36. 10.1016/j.shpsc.2011.10.003 22326070

[ref53] Leonelli S Ankeny RA : Repertoires: How to Transform a Project into a Research Community. *BioScience.* 2015;65(7):701–708. 10.1093/biosci/biv061 26412866 PMC4580990

[ref54] Leonelli S Davey R Arnaud E : Data Management and Best Practice in Plant Science. *Nat Plants.* 2017;3:17086. 10.1038/nplants.2017.86 28585570

[ref55] Levins R : The Strategy of Model Building in Population Biology. *Am Sci.* 1966;54(4):421–31.

[ref56] Lin : The TRUST Principles for Digital Repositories. *Scientific Data.* 2020. 10.1038/s41597-020-0486-7 PMC7224370 32409645

[ref57] Macdonald AJ : ed. Guide to the Classical and Other Long-Term Experiments. In: *Datasets and Sample Archive.* Harpenden: Rothamsted Research;2018. 10.23637/ROTHAMSTED-LONG-TERM-EXPERIMENTS-GUIDE-2018

[ref58] Mackenzie A Waterton C Ellis R : Classifying, Constructing, and Identifying Life: Standards as Transformations of ‘The Biological.’ *Science, Technology & Human Values.* 2013;38(5):701–22. 10.1177/0162243912474324

[ref59] Marx V : The big challenges of big data. *Nature.* 2013;498:255–260. 10.1038/498255a 23765498

[ref60] Minervini M Giuffrida MV Perata P : Phenotiki: an open software and hardware platform for affordable and easy image-based phenotyping of rosette-shaped plants. *Plant J.* 2017;90(1):204–216. 10.1111/tpj.13472 28066963

[ref61] Mitchell T : *Machine Learning.* New York: McGraw Hill;1997.

[ref62] Mochida K Koda S Inoue K : Statistical and Machine Learning Approaches to Predict Gene Regulatory Networks from Transcriptome Datasets. * Front Plant Sci.* 2018;9:1770. 10.3389/fpls.2018.01770 30555503 PMC6281826

[ref63] Mohanty SP Hughes DP Salathé M : Using Deep Learning for Image-Based Plant Disease Detection. *Front Plant Sci.* 2016;7:1419. 10.3389/fpls.2016.01419 27713752 PMC5032846

[ref64] Murtagh MJ Demir I Jenkings KN : Securing the Data Economy: Translating Privacy and Enacting Security in the Development of DataSHIELD. *Public Health Genomics.* 2012;15:243–253. 10.1159/000336673 22722688

[ref65] Napoletani D Panza M Struppa DC : Agnostic science. Towards a philosophy of data analysis. *Foundations of Science.* 2015;16(1):1–20. 10.1007/s10699-010-9186-7

[ref66] Natarajan N Dhillon IS Ravikumar PK Learning with Noisy Labels. In: Burges CJC Bottou L Welling M *Advances in Neural Information Processing Systems 26.* 2013. Reference Source

[ref67] Neveu P Tireau A Hilgert N : Dealing with multi-source and multi-scale information in plant phenomics: the ontology-driven Phenotyping Hybrid Information System. *New Phytol.* 2019;221:588–601. 10.1111/nph.15385 30152011 PMC6585972

[ref68] November J : *Biomedical Computing: Digitizing Life in the United States.* Baltimore: Johns Hopkins University Press;2012.

[ref69] Open Research Data Taskforce: Realising the potential: Final report of the Open Research Data Task Force. 2018. Reference Source

[ref70] Orr RJ Murray PJ Eyles CJ : The North Wyke Farm Platform: effect of temperate grasland farming systems on soil moisture contents, runoff and associated water quality dynamics. *Eur J Soil Sci.* 2016;67:374–385. 10.1111/ejss.12350 27867310 PMC5103177

[ref71] Papoutsoglou EA Faria D Arend D : Enabling reusability of plant phenomic datasets with MIAPPE 1.1. *New Phytol.* 2020;227(1):260–273. 10.1111/nph.16544 32171029 PMC7317793

[ref72] Park MG Blitzer EJ Gibbs J : Negative effects of pesticides on wild bee communities can be buffered by landscape context. *Proc Biol Sci.* 2015;282.1809:20150299. 10.1098/rspb.2015.0299 26041355 PMC4590442

[ref73] Parolini G : The Emergence of Modern Statistics in Agricultural Science: Analysis of Variance, Experimental Design and the Reshaping of Research at Rothamsted Experimental Station, 1919-1933. *J Hist Biol.* 2015;48:301–335. 10.1007/s10739-014-9394-z 25311906

[ref74] Perryman S Castells-Brooke N Glendining M : The electronic Rothamsted Archive (e-RA), an online resource for data from the Rothamsted long-term experiments. *Sci Data.* 2018;5:180072. 10.1038/sdata.2018.72 29762552 PMC5952867

[ref75] Pommier C Michotey C Cornut G : Applying FAIR Principles to Plant Phenotypic Data Management in GnpIS. *Plant Phenomics.* 2019;1671403. 10.34133/2019/1671403 33313522 PMC7718628

[ref76] Potamitis I Rigakis I Fysarakis K : Insect Biometrics: Optoacoustic Signal Processing and its Applications to Remote Monitoring of McPhail Type Traps. *PLOS ONE.* 2015;10(11):e0140474. 10.1371/journal.pone.0140474 26544845 PMC4636391

[ref77] Poulton P Johnston J Macdonald A : Major limitations to achieving “4 per 1000” increases in soil organic carbon stock in temperate regions: Evidence from long-term experiments at Rothamsted Research, United Kingdom. *Glob Chang Biol.* 2018;24(6):2563–2584. 10.1111/gcb.14066 29356243 PMC6001646

[ref78] Pound MP Atkinson JA Townsend AJ : Deep machine learning provides state-of-the-art performance in image-based plant phenotyping [published correction appears in Gigascience. 2018, 7(7)]. *Gigascience.* 2017;6(10):1–10. 10.1093/gigascience/gix083 29020747 PMC5632296

[ref79] Ramcharan A Baranowski K McCloskey P : Deep learning for image-based cassava disease detection. *Front Plant Sci.* 2017;8:1852. 10.3389/fpls.2017.01852 29163582 PMC5663696

[ref80] Ramcharan A McCloskey P Baranowski K : A Mobile-Based Deep Learning Model for Cassava Disease Diagnosis. *Front Plant Sci.* 2019;10:272. 10.3389/fpls.2019.00272 30949185 PMC6436463

[ref81] Reiser L Harper L Freeling M : FAIR: A Call to Make Published Data More Findable, Accessible, Interoperable, and Reusable. *Mol Plant.* 2018;11:1105–1108. 10.1016/j.molp.2018.07.005 30076986

[ref82] Rife TW Poland JA : Field Book: An Open-Source Application for Field Data Collection on Android. *Crop Sci.* 2014;54:1624–1627. 10.2135/cropsci2013.08.0579

[ref83] Rigden DJ Fernández XM : The 27th annual Nucleic Acids Research database issue and molecular biology database collection. *Nucleic Acids Res.* 2020;48(D1):D1–D8. 10.1093/nar/gkz1161 31906604 PMC6943072

[ref84] Roca T Letouze E : Open algorithms: A new paradigm for using private data for social good. *Devex.* 2016; (18 July). Reference Source

[ref85] Rodriguez-Iglesias A Rodriguez-Gonzalez A Irvine AG : Publishing FAIR Data: An Exemplar Methodology Using PHI-Base. *Front Plant Sci.* 2016; (12 May). 10.3389/fpls.2016.00641 27433158 PMC4922217

[ref86] Rosenqvist E Großkinsky DK Ottosen C-O : The Phenotyping Dilemma—The Challenges of a Diversified Phenotyping Community. *Front Plant Sci.* 2019;10:163. 10.3389/fpls.2019.00163 30873188 PMC6403123

[ref87] Schramowski P Stammer W Teso S : Making deep neural networks right for the right scientific reasons by interacting with their explanations. *Nature Machine Intelligence.* 2020;2:476–486. 10.1038/s42256-020-0212-3

[ref88] Selby P Abbeloos R Backlund JE : BrAPI—an application programming interface for plant breeding applications. *Bioinformatics.* 2019;31(20):4147–4155. 10.1093/bioinformatics/btz190 30903186 PMC6792114

[ref89] SEQC/MAQC-III Consortium: A comprehensive assessment of RNA-seq accuracy, reproducibility and information content by the Sequencing Quality Control Consortium. *Nat Biotechnol.* 2014;32:903–914. 10.1038/nbt.2957 25150838 PMC4321899

[ref90] Shaw F Etuk A Minotto A : COPO: a metadata platform for brokering FAIR data in the life sciences [version 1; peer review: 1 approved, 1 approved with reservations]. *F1000Research * 2020;9:495. 10.12688/f1000research.23889.1

[ref91] Singh A Ganapathysubramanian B Singh AK : Machine Learning for High-Throughput Stress Phenotyping in Plants. *Trends Plant Sci.* 2016;21(2):110–124. 10.1016/j.tplants.2015.10.015 26651918

[ref92] Smith G Cordes J : *Nine Pitfalls of Data Science.* Oxford: Oxford University Press;2019.

[ref93] Soltaninejad M Sturrock CJ Griffiths M : Three Dimensional Root CT Segmentation Using Multi-Resolution Encoder-Decoder Networks. *IEEE Trans Image Process.* 2020;29:6667–6679. 10.1109/TIP.2020.2992893 32406835

[ref94] Soltis PS : Digitization of herbaria enables novel research. *Am J Bot.* 2017;104(9):1281–1284. 10.3732/ajb.1700281 29885238

[ref95] Sperschneider J : Machine learning in plant-pathogen interactions: empowering biological predictions from field scale to genome scale. *New Phytol. Early view.* 2019. 10.1111/nph.15771 30834534

[ref96] Spindel JE McCouch SR : When more is better: how data sharing would accelerate genomic selection of crop plants. *New Phytol.* 2016;212(4):814–826. 10.1111/nph.14174 27716975

[ref97] Stanford NJ Wolstencroft K Golebiewski M : The evolution of standards and data management practices in systems biology. *Mol Syst Biol.* 2015;11:851. 10.15252/msb.20156053 26700851 PMC4704484

[ref98] Stevens H : *Life Out of Sequence: A Data-Driven History of Bioinformatics.* Chicago: Chicago University Press;2013.

[ref99] Strasser B : *Collecting Experiments: Making Big Data Biology.* Chicago: Chicago University Press;2019.

[ref100] Tardieu F Cabrera-Bosquet L Pridmore T : Plant Phenomics: From Sensors to Knowledge. *Curr Biol.* 2017;27(15):R770–R783. 10.1016/j.cub.2017.05.055 28787611

[ref101] Thiers BM : The World’s Herbaria 2020: A Summary Report Based on Data from Index Herbariorum. 2020. Reference Source

[ref103] Tiwari K Kananathan S Roberts MG : Reproducibility in systems biology modelling. *bioRxiv preprint.* 2020. 10.1101/2020.08.07.239855 PMC7901289 33620773

[ref104] Toda Y Okura F Ito J : Training instance segmentation neural network with synthetic datasets for crop seed phenotyping. *Commun Biol.* 2020;3:173. 10.1038/s42003-020-0905-5 32296118 PMC7160130

[ref105] Tsaftaris S Scharr H : Sharing the Right Data Right: A Symbiosis with Machine Learning. *Trends Plant Sci.* 2019;24(2):99–102. 10.1016/j.tplants.2018.10.016 30497879

[ref106] Tsiligiridis T Ainali K : Remote sensing Big AgriData for food availability. *Proc. SPIE 10836, 2018 International Conference on Image and Video Processing, and Artificial Intelligence, 108361G* 29 October 2018. 10.1117/12.2327014

[ref107] Ubbens J Cieslak M Prusinkiewicz P : The use of plant models in deep learning: an application to leaf counting in rosette plants. *Plant Methods.* 2018;14:6. 10.1186/s13007-018-0273-z 29375647 PMC5773030

[ref108] Van Beijma S Chatterton J Page S : The challenges of using satellite data sets to assess historical land use change and associated greenhouse gas emissions: a case study of three Indonesian provinces. *Carbon Management.* 2018;9:399–413. 10.1080/17583004.2018.1511383

[ref109] Wang H Cimen E Singh N : Deep Learning for Plant Genomics and Crop Improvements. *Curr Opin Plant Biol.* 2020;54:34–41. 10.1016/j.pbi.2019.12.010 31986354

[ref110] Wilkinson M Dumontier M Aalbersberg I : The FAIR Guiding Principles for scientific data management and stewardship. *Scientific Data.* 2018;3:160018. 10.1038/sdata.2016.18 PMC4792175 26978244

[ref115] Williamson HF Leonelli S (eds.): *Towards Responsible Plant Data Linkage: Data Challenges for Agricultural Research and Development.* Springer Open Access;2022.

[ref111] Wiseman L Sanderson J Robb L : Rethinking AgData Ownership. *Farm Policy J.* 2018;15(1):71–77.

[ref112] Yasrab R Atkinson JA Wels DM : RootNav 2.0: Deep learning for automatic navigation of complex plant root architectures. *GigaScience.* 2019;8(11):giz123. 10.1093/gigascience/giz123 31702012 PMC6839032

[ref113] Younis S Weiland C Hoehndorf R : Taxon and trait recognition from digitized herbarium specimens using deep convolutional neural networks. *Botany Letters.* 2018;165(3-4):377–383. 10.1080/23818107.2018.1446357

[ref114] Zou Q Sangaiah AK Mrozek D : Editorial: Machine Learning Techniques on Gene Function Prediction. *Front Genet.* 2019;10:938. 10.3389/fgene.2019.00938 31636657 PMC6788354

