# Supplementary material for: Data management challenges for artificial intelligence in plant and agricultural research
Source: F1000Res. 2023 Jan 17;10:324. Originally published 2021 Apr 27. [Version 2] doi: 10.12688/f1000research.52204.2 (PMC9975417; doi:10.12688/f1000research.52204.2)
Supplement: Peer review history

# Peer review: Reviewer response for version 2

**DOI:** 10.5256/f1000research.142478.r160522

**Published:** 2023-02-28

**Authors:** Daphne Ezer

**Affiliations:** 1 Department of Statistics, University of Warwick, Coventry, UK

I think that they have adequately addressed my concerns and would agree with the decision to Approve.

Is the topic of the opinion article discussed accurately in the context of the current literature?

Yes

Are arguments sufficiently supported by evidence from the published literature?

Yes

Are all factual statements correct and adequately supported by citations?

Yes

Are the conclusions drawn balanced and justified on the basis of the presented arguments?

Yes

Reviewer Expertise:

Machine learning in plant biology; transcriptomics

I confirm that I have read this submission and believe that I have an appropriate level of expertise to confirm that it is of an acceptable scientific standard.

# Peer review: Reviewer response for version 2

**DOI:** 10.5256/f1000research.142478.r160521

**Published:** 2023-01-18

**Authors:** Joeri Witteveen

**Affiliations:** 1 Department of Science Education, Section for History and Philosophy of Science, University of Copenhagen, Copenhagen, Denmark

The authors have adequately addressed my comments and suggestions. I have no further comments to make.

Is the topic of the opinion article discussed accurately in the context of the current literature?

Yes

Are arguments sufficiently supported by evidence from the published literature?

Yes

Are all factual statements correct and adequately supported by citations?

Yes

Are the conclusions drawn balanced and justified on the basis of the presented arguments?

Yes

Reviewer Expertise:

Philosophy of science

I confirm that I have read this submission and believe that I have an appropriate level of expertise to confirm that it is of an acceptable scientific standard.

# Peer review: Reviewer response for version 1

**DOI:** 10.5256/f1000research.55447.r154789

**Published:** 2022-11-14

**Authors:** Joeri Witteveen

**Affiliations:** 1 Department of Science Education, Section for History and Philosophy of Science, University of Copenhagen, Copenhagen, Denmark

This opinion article presents a solid overview of the state of the art of AI in plant research and provides a comprehensive analysis of current challenges in (and solution for) bringing AI approaches in plant research to full fruition.

 While each of the identified challenges is to the point, there is considerable overlap between them. This could have been avoided to some extent by embedding the discussion of these challenges in a clearer analytic framework, instead of by presenting them as more or less randomly ordered list. That said, this is a minor shortcoming and I don't think the article requires any major restructuring to prove its merits. Thus, I approve of the overall article in its current form and have only a few suggestions for minor corrections and further improvements:

 First, regarding the current status of the uses of AI in plant research, it would be helpful if the authors could expand their brief introduction of ML approaches by elaborating on which of these approaches are actually being used in plant research. It looks like the examples they go on to describe are all instances of (semi-)supervised learning. Are the other approaches to ML that they present also employed in plant science? If so, examples would be welcome. If not, it would be useful to signpost the more limited scope of AI approaches in plant science. Relatedly, I was slightly puzzled by the characterization of supervised learning as ‘a priori’ and of unsupervised learning as ‘inductive’. Both approaches are often considered inductive, and it is unclear why supervised learning should count as ‘a priori’. A final note on this point is that a reference to more recent literature (>1997) on ML would be appropriate here.

 Another suggestion for improvement concerns the relation between Table 1 and the main text. While the table as such provides a very useful summary of the challenges and solutions being discussed, I found that the contents of some of the cells does not adequately reflect the material from the main text. The characterization of challenge #5 in the ‘data challenges’ column is a case in point. In the table, this challenge is labeled “Access to computing and modeling platforms, and related expertise” whereas in the main text the corresponding subsection is entitled “Using software and models across scale, species and environment.” Only the first paragraph of this subsection discusses questions of accessibility. In the remainder, issues relating to the *use* of models across contexts and scales are the focus. This is one of several instances for which there appears to be an opportunity to ensure a better fit between the contents of the table and the main text.

 A further small issue about the table: the meaning of the contents in the “risks” column is ambiguous and inconsistent: in some rows, the risks pertain to the data challenge (when left unaddressed), whereas in others rows the risks pertain to the proposed solution. This should be easy to address, e.g. by introducing a further ‘tradeoffs’ column that lists the risks related to implementing the solutions (as opposed to leaving the challenges unaddressed).

 Finally, I was puzzled by this remark in the conclusion: “Once a machine learning strategy has been identified for a given task exposure to further examples of that task typically improves performance, even when the details and environment are significantly different.” This optimistic conclusion seemed to me to be somewhat at odds with the main message of the article. Unless we construe a ‘task’ in plant science artificially narrowly, it seems that that challenges that have been identified and discussed demonstrate that we encounter considerable difficulties in establishing reliable performance of ML across research contexts.

Is the topic of the opinion article discussed accurately in the context of the current literature?

Yes

Are arguments sufficiently supported by evidence from the published literature?

Yes

Are all factual statements correct and adequately supported by citations?

Yes

Are the conclusions drawn balanced and justified on the basis of the presented arguments?

Yes

Reviewer Expertise:

Philosophy of science

I confirm that I have read this submission and believe that I have an appropriate level of expertise to confirm that it is of an acceptable scientific standard.

# Response

**Published:** 2022-12-21

**Authors:** Sabina Leonelli

**Affiliations:** University of Exeter, UK

First, regarding the current status of the uses of AI in plant research, it would be helpful if the authors could expand their brief introduction of ML approaches by elaborating on which of these approaches are actually being used in plant research. It looks like the examples they go on to describe are all instances of (semi-)supervised learning. Are the other approaches to ML that they present also employed in plant science? If so, examples would be welcome. If not, it would be useful to signpost the more limited scope of AI approaches in plant science. Relatedly, I was slightly puzzled by the characterization of supervised learning as ‘a priori’ and of unsupervised learning as ‘inductive’. Both approaches are often considered inductive, and it is unclear why supervised learning should count as ‘a priori’. A final note on this point is that a reference to more recent literature (>1997) on ML would be appropriate here.

- *In response to reviewer 1’s comments, we have included an additional table (Table 2) documenting the examples of ML and AI applications discussed in the paper. As part of this table, we have also listed the key ML methods associated with those examples.*

Another suggestion for improvement concerns the relation between Table 1 and the main text. While the table as such provides a very useful summary of the challenges and solutions being discussed, I found that the contents of some of the cells does not adequately reflect the material from the main text. The characterization of challenge #5 in the ‘data challenges’ column is a case in point. In the table, this challenge is labeled “Access to computing and modeling platforms, and related expertise” whereas in the main text the corresponding subsection is entitled “Using software and models across scale, species and environment.” Only the first paragraph of this subsection discusses questions of accessibility. In the remainder, issues relating to the *use* of models across contexts and scales are the focus. This is one of several instances for which there appears to be an opportunity to ensure a better fit between the contents of the table and the main text.

- *Thank you for the useful comment, in some cases (such as that cited) this was a question of what we meant by ‘expertise’. We now improved the table to better match the contents of the paper. *

A further small issue about the table: the meaning of the contents in the “risks” column is ambiguous and inconsistent: in some rows, the risks pertain to the data challenge (when left unaddressed), whereas in others rows the risks pertain to the proposed solution. This should be easy to address, e.g. by introducing a further ‘tradeoffs’ column that lists the risks related to implementing the solutions (as opposed to leaving the challenges unaddressed).

- *Agreed and implemented, thank you. *

Finally, I was puzzled by this remark in the conclusion: “Once a machine learning strategy has been identified for a given task exposure to further examples of that task typically improves performance, even when the details and environment are significantly different.” This optimistic conclusion seemed to me to be somewhat at odds with the main message of the article. Unless we construe a ‘task’ in plant science artificially narrowly, it seems that that challenges that have been identified and discussed demonstrate that we encounter considerable difficulties in establishing reliable performance of ML across research contexts.

- *We corrected the sentence and added an additional sentence at the end of this paragraph to signal that this kind of success cannot always be expected or generalised. Real challenges remain, as identified in this paper, but addressing the challenges as suggested here does help to develop effective, targeted uses of ML (what the referee rightly labels a ‘narrow task’). *

# Peer review: Reviewer response for version 1

**DOI:** 10.5256/f1000research.55447.r95850

**Published:** 2021-10-18

**Authors:** Daphne Ezer

**Affiliations:** 1 Department of Statistics, University of Warwick, Coventry, UK

This is a good summary of data management and curation challenges in the plant science community. Initially, I was a bit skeptical for the need of this article, because the data management challenges listed were the same as those that are required across biology and across both data analysis AND AI. After reading this more carefully, I do think that this is useful, but mostly because of the list and description of excellent examples and case studies across each of the challenge areas addressed by the article.

 I would recommend that they maybe make a supplementary table of these examples for ease of reference. 

 In addition, I would be a bit careful about the definition of AI. I generally agree, but some people have strong feelings that ML and AI are distinct, because ML requires some level of manual feature extraction and so may be considered more of a set of statistical method than an "artificial intelligence". For instance, it is easy to argue that linear regression is ML, but very few people would call that AI. You use HMMs as an example of AI, which I think would be considered quite controversial. I would acknowledge that this is an area of controversy, but then stick with the definition you've set here.

 The biggest area that I think you need to be careful about though is the distinction between labelling individual "data points" (such as individual images) and metadata about the overall dataset. Metadata about the experimental conditions, species, etc is often easier to come by (and already required by many data repositories), but adding labels to enough data points to enable supervised learning is an order of magnitude harder. You address this when you talk about reliability, but in other places these concepts are slightly conflated. 

 One issue with data management for AI is that there is often a lot of data collected about a single or small amount of observations (one species, one or two experimental conditions, etc). However, this kind of thing isn't suitable for machine learning/AI, since AI relies on there being a large number of observations. 

 Another issue is the lack of standardisation of experimental profiles. Even if all the meta-information were present, it would still be incredibly challenging to extract batch effects from experiments across labs, given the number of variables that people are changing. Do the authors really believe that making the data FAIR and including metadata would help overcome this problem? It seems like doing a meta-analysis of data across labs using AI will always be somewhat problematic, even if the data was all FAIR.

Is the topic of the opinion article discussed accurately in the context of the current literature?

Yes

Are arguments sufficiently supported by evidence from the published literature?

Yes

Are all factual statements correct and adequately supported by citations?

Yes

Are the conclusions drawn balanced and justified on the basis of the presented arguments?

Yes

Reviewer Expertise:

Machine learning in plant biology; transcriptomics

I confirm that I have read this submission and believe that I have an appropriate level of expertise to confirm that it is of an acceptable scientific standard, however I have significant reservations, as outlined above.

# Response

**Published:** 2022-12-21

**Authors:** Sabina Leonelli

**Affiliations:** University of Exeter, UK

This is a good summary of data management and curation challenges in the plant science community. Initially, I was a bit skeptical for the need of this article, because the data management challenges listed were the same as those that are required across biology and across both data analysis AND AI. After reading this more carefully, I do think that this is useful, but mostly because of the list and description of excellent examples and case studies across each of the challenge areas addressed by the article.

 I would recommend that they maybe make a supplementary table of these examples for ease of reference.

- *We have added a supplementary table listing the major examples of machine learning and AI discussed in the paper. We have not included examples of databases where the potential for application of ML/AI methods has been discussed, but no work has yet been attempted or published.*

In addition, I would be a bit careful about the definition of AI. I generally agree, but some people have strong feelings that ML and AI are distinct, because ML requires some level of manual feature extraction and so may be considered more of a set of statistical method than an "artificial intelligence". For instance, it is easy to argue that linear regression is ML, but very few people would call that AI. You use HMMs as an example of AI, which I think would be considered quite controversial. I would acknowledge that this is an area of controversy, but then stick with the definition you've set here.

- *We have added a footnote in section 2 recognising that there is disagreement over the classification of ML as AI.*

The biggest area that I think you need to be careful about though is the distinction between labelling individual "data points" (such as individual images) and metadata about the overall dataset. Metadata about the experimental conditions, species, etc is often easier to come by (and already required by many data repositories), but adding labels to enough data points to enable supervised learning is an order of magnitude harder. You address this when you talk about reliability, but in other places these concepts are slightly conflated.

- *We have added an additional comment at the start of section 3.4 on the differences between labelling metadata and labelling individual data points. In subsequent sections we have clarified where the discussion concerns labelling of metadata and labelling/annotating individual data points, especially in section 3.6 where the discussion shifts from data annotation to errors in metadata.*

One issue with data management for AI is that there is often a lot of data collected about a single or small amount of observations (one species, one or two experimental conditions, etc). However, this kind of thing isn't suitable for machine learning/AI, since AI relies on there being a large number of observations.

- *The whole paper concerns attempts to pool datasets (large and small), so we agree with the idea that very small datasets are not amenable to AI approaches, but this is not what we are discussing here. We clarified this further in the paper. *

Another issue is the lack of standardisation of experimental profiles. Even if all the meta-information were present, it would still be incredibly challenging to extract batch effects from experiments across labs, given the number of variables that people are changing. Do the authors really believe that making the data FAIR and including metadata would help overcome this problem? It seems like doing a meta-analysis of data across labs using AI will always be somewhat problematic, even if the data was all FAIR.

- *We agree that this challenge remains, and have inserted a paragraph in the conclusion of the paper (right after the suggestions that all data should be FAIR) pointing this out. Many thanks for this and the above suggestions!*
